# Topical Anti‐Inflammatory Treatments for Eczema: A Cochrane Systematic Review and Network Meta‐Analysis

**DOI:** 10.1111/cea.14556

**Published:** 2024-09-02

**Authors:** Stephanie J. Lax, Eleanor Van Vogt, Bridget Candy, Lloyd Steele, Clare Reynolds, Beth Stuart, Roses Parker, Emma Axon, Amanda Roberts, Megan Doyle, Derek K. Chu, Masaki Futamura, Miriam Santer, Hywel C. Williams, Suzie Cro, Aaron M. Drucker, Robert J. Boyle

**Affiliations:** ^1^ Centre of Evidence Based Dermatology University of Nottingham Nottingham UK; ^2^ Imperial Clinical Trials Unit Imperial College London London UK; ^3^ Department of Dermatology Royal Free Hospital London UK; ^4^ Wellcome Sanger Institute Cambridge UK; ^5^ School of Public Health, Physiotherapy and Sports Science, University College Dublin Dublin Ireland; ^6^ Primary Care and Population Sciences, Faculty of Medicine University of Southampton Southampton UK; ^7^ Cochrane MOSS Network, c/o Cochrane Pain Palliative and Supportive Care Group Oxford UK; ^8^ Cochrane Methods Support Unit Cochrane London UK; ^9^ Nottingham Support Group for Carers of Children With Eczema Nottingham UK; ^10^ Department of Medicine McMaster University Hamilton Ontario Canada; ^11^ Department of Health Research Methods, Evidence & Impact McMaster University Hamilton Ontario Canada; ^12^ Department of Pediatrics National Hospital Organization Nagoya Medical Center Nagoya Japan; ^13^ Department of Medicine University of Toronto Toronto Ontario Canada; ^14^ Department of Medicine, Research and Innovation Institute Women's College Hospital Toronto Ontario Canada; ^15^ Section of Inflammation and Repair, National Heart & Lung Institute Imperial College London London UK

**Keywords:** calcineurin inhibitor, eczema, Janus kinase inhibitor, network meta‐analysis, systematic review, topical steroid

## Abstract

**Objective:**

Eczema is the most burdensome skin condition worldwide and topical anti‐inflammatory treatments are commonly used to control symptoms. The relative effectiveness and safety of different topical anti‐inflammatory treatments is uncertain.

**Design:**

Network meta‐analysis performed within a Cochrane systematic review to compare and statistically rank efficacy and safety of topical anti‐inflammatory eczema treatments.

**Data Sources:**

Cochrane Skin Specialised Register, CENTRAL, MEDLINE, Embase and trial registries to June 2023.

**Eligibility Criteria for Selected Trials:**

Included trials were within‐participant or between‐participant randomised controlled trials. Participants had eczema that was not clinically infected and was not contact dermatitis, seborrheic eczema or hand eczema. Interventions were topical anti‐inflammatory treatments but not complementary treatments, antibiotics alone, wet wraps, phototherapy or systemic treatments. Comparators were no treatment/vehicle or another topical anti‐inflammatory.

**Results:**

We identified 291 trials (45,846 participants), mainly in high‐income countries. Most were industry‐funded with median 3 weeks treatment duration. Risk of bias assessed using the Cochrane Risk of Bias 2.0 tool was high in 89% of trials, mainly due to risk of selective reporting. Network meta‐analysis of binary outcomes ranked potent and/or very potent topical steroids, tacrolimus 0.1% and ruxolitinib 1.5% among the most effective treatments for improving patient‐reported symptoms (40 trials, all low confidence) and clinician‐reported signs (32 trials, all moderate confidence). For investigator global assessment, the Janus kinas inhibitors ruxolitinib 1.5%, delgocitinib 0.5% or 0.25%, very potent/potent topical steroids and tacrolimus 0.1% were ranked as most effective (140 trials, all moderate confidence). Continuous outcome data were mixed. Local application site reactions were most common with tacrolimus 0.1% (moderate confidence) and crisaborole 2% (high confidence) and least common with topical steroids (moderate confidence). Skin thinning was not increased with short‐term use of any topical steroid potency (low confidence) but skin thinning was reported in 6/2044 (0.3%) participants treated with longer‐term (6–60 months) topical steroids.

**Conclusion:**

Potent topical steroids, Janus kinase inhibitors and tacrolimus 0.1% were consistently ranked as among the most effective topical anti‐inflammatory treatments for eczema.

## Graphical abstract

Trials of topical anti‐inflammatory eczema treatments are mostly industry‐funded, evaluate short‐term outcomes and carry a high risk of bias due to selective reporting. Data from almost 300 trials suggest that potent steroids, Janus kinase inhibitors and tacrolimus 0.1% are among the most effective topical treatments. Local reactions were most common with tacrolimus 0.1% and crisaborole and least common with steroids.

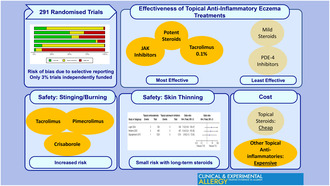

**Summary**

- Trials of topical anti‐inflammatory eczema treatments are mostly industry‐funded, short‐term and high risk of bias.
- Potent steroids, Janus kinase inhibitors and tacrolimus 0.1% were among the most effective topical treatments.
- Local reactions were most common with tacrolimus 0.1% and crisaborole and least common with steroids.

## 1 Introduction

Eczema affects up to 20% of infants, 6% of school‐age children and 5% of adults worldwide and is the most burdensome skin condition globally [1, 2, 3]. Topical corticosteroids (TCS) are the most commonly used anti‐inflammatory treatment for eczema and have been available for over 70 years. While there is great interest in novel systemic treatments for moderate–severe eczema, new classes of topical anti‐inflammatory treatments have also been licensed for mild, moderate or severe eczema treatment [4, 5]. Topical calcineurin inhibitors (TCI) tacrolimus and pimecrolimus were licensed in 2000, and subsequently phosphodiesterase 4 (PDE‐4) inhibitors such as crisaborole, Janus kinase (JAK) inhibitors such as ruxolitinib and aryl hydrocarbon receptor activators such as tapinarof have subsequently become available or are in development. There is a lack of comparative effectiveness research in eczema, as in many other areas of healthcare [6, 7]. This paucity makes it very difficult for health care professionals and patients to decide which treatment is best in terms of benefits and the least harms. It also hampers guideline development. Network meta‐analysis (NMA) is a tool developed for indirectly evaluating the relative effectiveness and safety of interventions used to treat the same condition. One NMA that included different interventions in addition to topical anti‐inflammatory treatments was published after our work started [8].

We conducted a NMA focussed on use of topical anti‐inflammatory treatments for treating eczema, in order to statistically rank and compare the effectiveness and safety of different topical anti‐inflammatory treatments.

## 2 Materials and Methods

This systematic review and NMA was conducted using standard Cochrane methodology, and according to its own pre‐published protocol and statistical analysis plan [9]. In brief, we included within‐participant or between‐participant randomised controlled trials (RCTs). Eligible trials evaluated people of any age with a clinical diagnosis of eczema (also called atopic dermatitis or atopic eczema) of any severity [10]. Specific, non‐atopic forms of eczema such as contact dermatitis, hand eczema or seborrheic eczema and clinically infected eczema were excluded. Eligible interventions included any well‐characterised topical anti‐inflammatory treatment. Emollients alone, topical antibiotics alone, complementary therapies, wet wraps, systemic treatment and phototherapy were excluded. In general, non‐licensed or non‐standard treatment regimens were excluded—for example application less than once daily or more than twice daily, or treatment durations of under 1 week. Comparison was to other topical anti‐inflammatory treatments, placebo/vehicle/emollient or no treatment. TCS were classified as mild, moderate, potent or very potent, as previously described [11]. Outcomes and outcome measures were prioritised according to the Harmonising Outcome Measures for Eczema (HOME) initiative [12, 13]; and informed by patient and public involvement during preliminary surveys and workshops as part of trial protocol development. Outcomes evaluated are summarised below:

### 2.1 Primary Outcomes

1. Patient‐reported symptoms of eczema. Data were extracted based on the Patient‐Oriented Eczema Measure (POEM) or, if not available, alternative instruments ranked in order of preference in the protocol, most commonly a visual analogue scale of pruritus [9].
2. Clinician‐reported signs of eczema. Data were extracted based on the Eczema Area and Severity Index [14] or, if not available, alternative instruments ranked in order of preference in the protocol, most commonly the Scoring Atopic Dermatitis [15]. Investigator Global Assessment (IGA) was considered separately from other clinician‐reported signs of eczema, since the construct of IGA as a single global assessment is emphasised by some regulatory authorities and may differ from other measures of eczema signs.

### 2.2 Secondary Outcomes

1. Health‐related quality of life. Data were extracted based on the Dermatology Life Quality Index including children's and infants' versions [16], or, if not available alternative instruments ranked in order of preference in the protocol.
2. Long‐term control of eczema. Data were extracted based on the Recap of Eczema Control [17] or, if not available, alternative instruments such as the Atopic Dermatitis Control Tool [18].
3. Local adverse effects. These were prioritised in patient and public involvement work as local application site reactions (‘tolerability’), cosmetic effects such as pigmentation changes, skin thinning/atrophy and withdrawal from treatment or trial due to adverse effects of the intervention.

### 2.3 Search Strategy

We searched the Cochrane Skin Specialised Register, CENTRAL, MEDLINE, Embase, the World Health Organization clinical trial meta‐registry and clinicaltrials.gov up to June 2023. The full search strategy is shown in the Cochrane review protocol [9].

### 2.4 Data Collection and Analysis

This was a frequentist NMA undertaken according to the methods of the Cochrane Handbook for Systematic Reviews of Interventions Version 6.2 [19]. Analysis was conducted following a statistical analysis plan with planned networks, sensitivity and subgroup analyses which were described in the protocol and finalised before undertaking data analysis. Networks were planned for patient‐reported symptoms and clinician assessed signs of eczema, separately for binary and continuous outcomes and for short and long‐term outcomes. IGA was treated separately with its own network(s) and safety outcomes were analysed with separate networks for application site reactions, pigmentation changes, skin thinning/atrophy and withdrawals due to adverse events. For long‐term outcomes and for quality of life there was insufficient data available to create networks. NMA was performed using a random‐effects model summarising either odds ratios (ORs) or standardised mean differences (SMDs) in Stata with the mvmeta command within the network suite of commands [20] and the Stata commands for graphing, statistically ranking using the Surface Under the Cumulative Ranking (SUCRA) score and reporting network results [21]. Heterogeneity was quantified using the heterogeneity parameter Tau. Local consistency was evaluated using the node‐splitting approach with Stata's sidesplit command [20] and global design inconsistency was evaluated using the ‘design by treatment interaction’ model [22]. Risk of bias of included trials was assessed using the Cochrane risk of bias 2.0 tool [23]. Certainty of evidence was assessed using the Confidence In NEtwork Meta‐Analysis (CINEMA) approach [24]. We planned for primary analyses to include only low risk of bias data. However, only one network was possible due to a lack of low risk of bias data, so we undertook primary analyses on ‘all available data’ and a sensitivity analysis, where possible, for the low risk of bias data. Other sensitivity analyses used alternative classification for TCS potency, restricted outcome data to HOME‐recommended measurement tools and excluding within‐participant data. Class‐level sensitivity analyses were also undertaken. Subgroup analyses explored potential impact of application site (face included versus not included), disease severity (severe versus non‐severe) and age (under 12 years or older). Summary of Findings Tables were created for each outcome where NMA was possible, and included only topical anti‐inflammatory interventions which are currently licensed, applied using the licensed concentration(s). Additional methods are shown in the Cochrane review protocol [9].

## 3 Results

**FIGURE 1:**
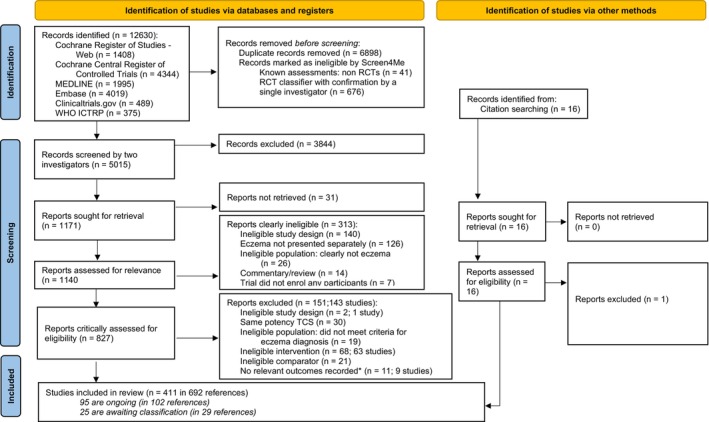
PRISMA flow diagram. *These were immunohistochemical analyses (*n* = 4), skin barrier analyses (*n* = 2), cost‐effectiveness analyses (*n* = 1) or additional records to existing trials with no new relevant outcome data (*n* = 4 from 2 trials). All were checked for effectiveness or safety outcomes.

**FIGURE 2:**
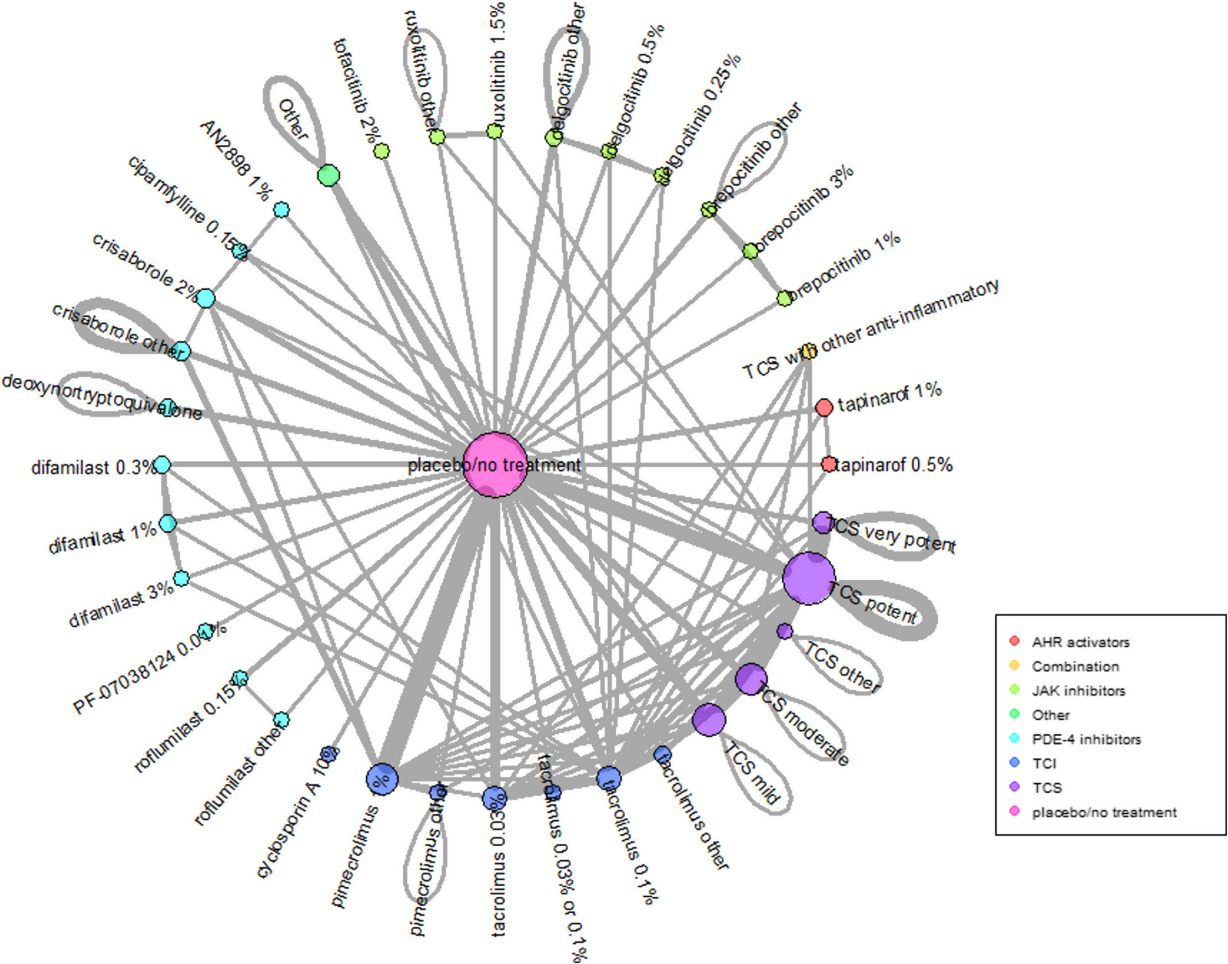
Network of included interventions. AHR, aryl hydrocarbon, JAK, Janus kinase, PDE‐4, phosphodiesterase‐4, TCI, topical calcineurin inhibitor, TCS, topical corticosteroid. Network shows all interventions included in the 291 included trials. Lines represent comparisons made in the included trials. The thickness of each line represents the number of separate trials making the comparison. This network represents all interventions, but the Summary of Findings Tables report only licensed interventions at licensed doses/concentrations.

**FIGURE 3:**
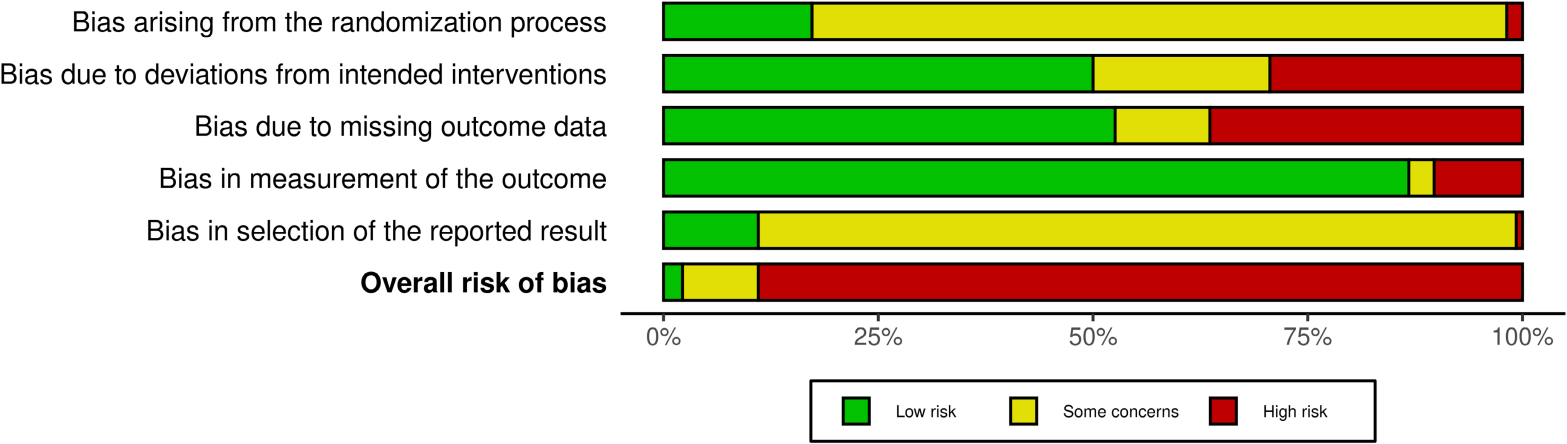
Summary of risk of bias in included trials. Summary Cochrane Risk of bias 2.0 assessments of the 291 included trials. For trials reporting multiple outcomes, the lowest risk of bias outcome is included in this summary.

Search results are summarised in Figure 1 and the review is reported according to the PRISMA statement and NMA extension [25, 26]. We identified 291 trials (45,846 participants) with published outcomes with the full spectrum of eczema severity and a further 120 trials which were either ongoing (95) or couldn't be classified (25). Trials were mainly conducted in high‐income countries (243) especially Europe and North America, and in secondary care settings (189). Adults were included in most trials, with only 31 trials limited to children aged <12 years. Male and female participants and multiple ethnic groups were present in most trials, but trials were mainly undertaken in predominantly white populations. Ninety‐seven per cent of trials were either industry‐funded (199) or did not report their funding (85). Treatment duration and trial participation were median 21 and 28 days (range 7 days to 5 years). Interventions used in the included trials are summarised in Figure 2. They were TCS (172), TCI (134), PDE‐4 inhibitors (55), JAK inhibitors (30), aryl hydrocarbon receptor activators (10) or other topical agents (21). Comparators included vehicle (170) or other anti‐inflammatory treatments. Risk of bias is summarised in Figure 3. Using the outcome with lowest risk of bias from each trial, risk of bias was high in 242 of the 272 (89%) trials contributing to data analyses. The most common risk of bias issue was concern about selective reporting due to absence of prospective trial registration/protocol availability, even in more recent trials. Other issues noted were insufficient information to judge allocation concealment, concern about contamination in within‐participant trials, poor reporting of numbers of randomised participants included in outcome analysis, exclusions from analysis for potentially inappropriate reasons such as adverse events and trials with high proportions of randomised participants missing from analyses.

The pooled direct and indirect effects of licensed interventions compared with placebo are summarised for each outcome in the Summary of Findings Tables (Tables 1, 2, 3, 4, 5), including CINeMA certainty of evidence ratings.

**TABLE 1:** Summary of findings for patient‐reported symptoms (binary).

Treatment	Direct evidence 40 RCTs, 6482 participants	Relative effect OR (95% CI)	Anticipated response rate with control	Anticipated response rate with treatment	Difference (95% CI)	SUCRA	CINeMA
TCS potent	6 RCTs, 699 participants	5.99 (2.83, 12.69)	309 per 1000	729 per 1000	420 per 1000 (250 to 541)	0.75	Low
Tacrolimus 0.1%	Indirect only	6.27 (1.19, 32.98)	309 per 1000	738 per 1000	429 per 1000 (39 to 627)	0.74	Low
Ruxolitinib 1.5%	2 RCTs, 465 participants	5.64 (1.26, 25.25)	309 per 1000	716 per 1000	407 per 1000 (51 to 609)	0.71	Low
TCS very potent	1 RCTs, 61 participants	5.08 (0.51, 50.76)	309 per 1000	695 per 1000	386 per 1000 (−124 to 648)	0.65	Low
Tacrolimus 0.03%	Indirect only	4.56 (0.49, 42.48)	309 per 1000	671 per 1000	362 per 1000 (−130 to 641)	0.63	Low
Pimecrolimus 1%	10 RCTs, 1712 participants	3.59 (1.84, 7.01)	309 per 1000	617 per 1000	308 per 1000 (142 to 449)	0.58	Low
Tapinarof 1%	1 RCTs, 77 participants	3.13 (0.33, 29.34)	309 per 1000	583 per 1000	274 per 1000 (−180 to 620)	0.52	Low
TCS moderate	5 RCTs, 1426 participants	2.62 (1.18, 5.81)	309 per 1000	540 per 1000	231 per 1000 (37 to 413)	0.47	Low
Crisaborole 2%	1 RCTs, 63 participants	1.15 (0.17, 7.71)	309 per 1000	341 per 1000	32 per 1000 (−238 to 466)	0.27	Low
Roflumilast 0.15%	1 RCTs, 81 participants	1.03 (0.12, 9.23)	309 per 1000	316 per 1000	7 per 1000 (−260 to 496)	0.26	Moderate
TCS mild	2 RCTs, 80 participants	1.35 (0.51, 3.53)	309 per 1000	376 per 1000	67 per 1000 (−122 to 303)	0.25	Low

*Note:* Trials compared topical anti‐inflammatory treatments to vehicle in people with eczema, and evaluated patient‐reported symptoms as a binary measure at 1–16 weeks after treatment initiation. Most trials were undertaken in a secondary care setting. A ≥ 4 point improvement in the Peak Pruritus Numerical Rating Scale was the most commonly reported measure.

Abbreviations: CI, confidence interval; CINeMA, confidence in meta‐analysis, OR, odds ratio; RCT, randomised controlled trial; SUCRA, surface area under the cumulative ranking curve; TCS, topical corticosteroid.

**TABLE 2:** Summary of findings for clinician‐reported signs (binary).

Treatment	Direct evidence 32 RCTs, 4121 participants	Relative effect OR (95% CI)	Anticipated response rate with control	Anticipated response rate with treatment	Difference (95% CI)	SUCRA	CINeMA
TCS potent	4 RCTs, 341 participants	8.15 (4.90, 13.57)	191 per 1000	658 per 1000	467 per 1000 (345 to 571)	0.87	Moderate
Tacrolimus 0.1%	1 RCTs, 61 participants	8.06 (3.30, 19.67)	191 per 1000	655 per 1000	464 per 1000 (247 to 632)	0.84	Moderate
Ruxolitinib 1.5%	3 RCTs, 879 participants	7.72 (4.92, 12.10)	191 per 1000	645 per 1000	454 per 1000 (346 to 550)	0.84	Moderate
Delgocitinib 0.5%	3 RCTs, 323 participants	7.61 (3.72, 15.58)	191 per 1000	642 per 1000	451 per 1000 (276 to 595)	0.83	Moderate
Difamilast 1%	2 RCTs, 532 participants	5.42 (3.06, 9.58)	191 per 1000	561 per 1000	370 per 1000 (228 to 502)	0.67	Moderate
Delgocitinib 0.25%	3 RCTs, 306 participants	5.26 (2.55, 10.87)	191 per 1000	554 per 1000	363 per 1000 (185 to 528)	0.65	Moderate
TCS moderate	1 RCTs, 37 participants	5.22 (2.55, 10.67)	191 per 1000	552 per 1000	361 per 1000 (185 to 525)	0.64	Moderate
Pimecrolimus 1%	5 RCTs, 750 participants	3.65 (2.40, 5.57)	191 per 1000	463 per 1000	272 per 1000 (170 to 377)	0.48	Moderate
Difamilast 0.3%	1 RCTs, 166 participants	3.22 (1.45, 7.13)	191 per 1000	431 per 1000	240 per 1000 (64 to 436)	0.42	High
Crisaborole 2%	2 RCTs, 169 participants	2.98 (1.42, 6.26)	191 per 1000	413 per 1000	222 per 1000 (60 to 405)	0.39	High
Roflumilast 0.15%	1 RCTs, 89 participants	2.43 (0.88, 6.70)	191 per 1000	364 per 1000	173 per 1000 (−19 to 422)	0.33	Very Low
Tapinarof 1%	1 RCTs, 126 participants	2.45 (1.00, 6.02)	191 per 1000	366 per 1000	175 per 1000 (−1 to 396)	0.32	Low
TCS mild	1 RCTs, 44 participants	2.22 (0.74, 6.64)	191 per 1000	343 per 1000	152 per 1000 (−42 to 419)	0.28	Low

*Note:* Trials compared topical anti‐inflammatory treatments to vehicle in people with eczema, and evaluated clinician‐reported signs as a binary measure at 1–16 weeks after treatment initiation. Most trials were undertaken in a secondary care setting. A ≥75% improvement in Eczema Area and Severity Index was the most commonly reported measure.

Abbreviations: CI, confidence interval; CINeMA, confidence in meta‐analysis, OR, odds ratio; RCT, randomised controlled trial; SUCRA, surface area under the cumulative ranking curve; TCS, topical corticosteroid.

**TABLE 3:** Summary of findings for Investigator Global Assessment (binary).

Treatment	Direct evidence 140 RCTs, 23,383 participants	Relative effect OR (95% CI)	Anticipated response rate with control	Anticipated response rate with treatment	Difference (95% CI)	SUCRA	CINeMA
Ruxolitinib 1.5%	3 RCTs, 879 participants	9.34 (4.80, 18.18)	256 per 1000	762 per 1000	506 per 1000 (367 to 606)	0.86	Moderate
TCS very potent	2 RCTs, 438 participants	8.34 (4.73, 14.67)	256 per 1000	741 per 1000	485 per 1000 (364 to 579)	0.84	Moderate
Delgocitinib 0.5%	2 RCTs, 165 participants	10.08 (2.65, 38.37)	256 per 1000	776 per 1000	520 per 1000 (221 to 674)	0.83	Moderate
Delgocitinib 0.25%	2 RCTs, 169 participants	6.87 (1.79, 26.33)	256 per 1000	702 per 1000	446 per 1000 (126 to 645)	0.73	Moderate
Tacrolimus 0.1%	10 RCTs, 1718 participants	5.06 (3.59, 7.13)	256 per 1000	635 per 1000	379 per 1000 (297 to 454)	0.68	Moderate
TCS potent	16 RCTs, 1708 participants	5 (3.80, 6.58)	256 per 1000	632 per 1000	376 per 1000 (310 to 438)	0.67	Moderate
TCS moderate	8 RCTs, 1335 participants	4.46 (3.19, 6.24)	256 per 1000	605 per 1000	349 per 1000 (267 to 426)	0.62	Moderate
Tapinarof 1%	3 RCTs, 262 participants	3.68 (1.73, 7.82)	256 per 1000	558 per 1000	302 per 1000 (117 to 473)	0.53	Moderate
Difamilast 1%	6 RCTs, 927 participants	3.45 (1.97, 6.02)	256 per 1000	542 per 1000	286 per 1000 (148 to 418)	0.51	Moderate
Tacrolimus 0.03%	10 RCTs, 2576 participants	3.53 (2.60, 4.80)	256 per 1000	548 per 1000	292 per 1000 (216 to 367)	0.51	Moderate
Roflumilast 0.15%	1 RCTs, 89 participants	2.43 (0.65, 9.01)	256 per 1000	454 per 1000	198 per 1000 (−73 to 500)	0.39	Moderate
Difamilast 0.3%	5 RCTs, 558 participants	2.56 (1.37, 4.78)	256 per 1000	468 per 1000	212 per 1000 (65 to 366)	0.38	Moderate
Pimecrolimus 1%	17 RCTs, 4064 participants	2.39 (1.78, 3.21)	256 per 1000	451 per 1000	195 per 1000 (123 to 269)	0.35	Moderate
Crisaborole 2%	5 RCTs, 1725 participants	2.14 (1.22, 3.76)	256 per 1000	424 per 1000	168 per 1000 (40 to 308)	0.32	Low
TCS mild	1 RCTs, 46 participants	1.38 (0.94, 2.02)	256 per 1000	321 per 1000	65 per 1000 (−12 to 154)	0.17	Low

*Note:* Trials compared topical anti‐inflammatory treatments to vehicle in people with eczema, and evaluated investigator global assessment as a binary measure at 1–16 weeks after treatment initiation. Most trials were undertaken in a secondary care setting. ‘Clear or almost clear’ eczema on a 6‐point Investigator Global Assessment was the most commonly reported measure.

Abbreviations: CI, confidence interval; CINeMA, confidence in meta‐analysis, OR, odds ratio; RCT, randomised controlled trial; SUCRA, surface area under the cumulative ranking curve; TCS, topical corticosteroid.

**TABLE 4:** Summary of findings for application site reactions (binary).

Treatment	Direct evidence 83 RCTs, 18,992 participants	Relative effect OR (95% CI)	Anticipated response rate with control	Anticipated response rate with treatment	Difference (95% CI)	SUCRA	CINeMA
Tacrolimus 0.1%	5 RCTs, 2364 participants	2.09 (1.46, 3.00)	80 per 1000	154 per 1000	74 per 1000 (33 to 126)	0.83	Moderate
Crisaborole 2%	4 RCTs, 1247 participants	2.11 (1.19, 3.76)	80 per 1000	155 per 1000	75 per 1000 (14 to 166)	0.82	High
Pimecrolimus 1%	15 RCTs, 2482 participants	1.49 (1.05, 2.12)	80 per 1000	114 per 1000	35 per 1000 (4 to 75)	0.71	Low
Tacrolimus 0.03%	8 RCTs, 3470 participants	1.49 (1.08, 2.04)	80 per 1000	114 per 1000	34 per 1000 (6 to 70)	0.71	Low
Tapinarof 1%	1 RCTs, 163 participants	1.01 (0.06, 17.91)	80 per 1000	81 per 1000	1 per 1000 (−75 to 528)	0.57	Low
TCS mild	1 RCTs, 768 participants	0.51 (0.30, 0.85)	80 per 1000	42 per 1000	−38 per 1000 (−54 to −11)	0.38	Moderate
TCS moderate	3 RCTs, 670 participants	0.49 (0.25, 0.93)	80 per 1000	40 per 1000	−39 per 1000 (−58 to −5)	0.37	Moderate
Roflumilast 0.15%	1 RCTs, 90 participants	0.33 (0.01, 8.84)	80 per 1000	27 per 1000	−52 per 1000 (−79 to 354)	0.32	Moderate
TCS potent	7 RCTs, 1149 participants	0.35 (0.22, 0.55)	80 per 1000	29 per 1000	−51 per 1000 (−61 to −34)	0.26	Moderate
TCS very potent	3 RCTs, 492 participants	0.33 (0.13, 0.81)	80 per 1000	27 per 1000	−52 per 1000 (−69 to −14)	0.25	Low

*Note:* Trials compared topical anti‐inflammatory treatments to vehicle in people with eczema, and evaluated investigator application site reactions as a binary measure at 1–16 weeks after treatment initiation. Most trials were undertaken in a secondary care setting. Application site reactions included tolerability events, burning, stinging and irritation.

Abbreviations: CI, confidence interval; CINeMA, confidence in meta‐analysis, OR, odds ratio; RCT, randomised controlled trial; SUCRA, surface area under the cumulative ranking curve; TCS, topical corticosteroid.

**TABLE 5:** Summary of findings for skin thinning/atrophy (binary).

Treatment	Direct evidence 83 RCTs, 18,992 participants	Relative effect OR (95% CI)	Anticipated response rate with control	Anticipated response rate with treatment	Difference (95% CI)	SUCRA	CINeMA
TCS potent	3 RCTs, 578 participants	0.96 (0.21, 4.43)	7 per 1000	7 per 1000	0 per 1000 (−6 to 24)	0.57	Low
TCS moderate	1 RCTs, 51 participants	0.91 (0.16, 5.33)	7 per 1000	7 per 1000	0 per 1000 (−6 to 30)	0.54	Low
TCS very potent	3 RCTs, 919 participants	0.88 (0.31, 2.49)	7 per 1000	6 per 1000	−1 per 1000 (−5 to 11)	0.52	Low
Tacrolimus 0.1%	indirect only	0.88 (0.01, 60.36)	7 per 1000	6 per 1000	−1 per 1000 (−7 to 299)	0.51	Low
TCS mild	1 RCTs, 768 participants	0.72 (0.12, 4.31)	7 per 1000	5 per 1000	−2 per 1000 (−6 to 23)	0.44	Low
Pimecrolimus 1%	1 RCTs, 38 participants	0.15 (0.01, 1.59)	7 per 1000	1 per 1000	−6 per 1000 (−7 to 4)	0.10	Low

*Note:* Trials compared topical anti‐inflammatory treatments to vehicle in people with eczema, and evaluated skin thinning/striae as a binary measure at 1–16 weeks after treatment initiation. Most trials were undertaken in a secondary care setting. Skin thinning/striae included skin thinning, atrophy, striae or telangiectasia.

Abbreviations: CI, confidence interval; CINeMA, confidence in meta‐analysis, OR, odds ratio; RCT, randomised controlled trial; SUCRA, surface area under the cumulative ranking curve; TCS, topical corticosteroid.

### 3.1 Patient‐Reported Eczema Symptoms

NMA included 40 trials (*n* = 6482) which most commonly reported a ≥4 point improvement in the Peak Pruritus Numerical Rating Scale. Potent TCS (OR 5.99, 95% CI 2.83, 12.69), the TCI tacrolimus 0.1% (OR 6.27, 95% CI 1.19, 32.98) and the JAK inhibitor ruxolitinib 1.5% (OR 5.64, 95% CI 1.26, 25.25) were ranked as most effective. Mild TCS (OR 1.35, 95% CI 0.51, 3.53) and the PDE‐4 inhibitors roflumilast 0.15% (OR 1.03, 95% CI 0.12, 9.23) and crisaborole 2% (OR 1.15, 95% CI 0.17, 7.71) were ranked as least effective (Table 1). Confidence intervals were wide and overlapping for most comparisons, and CINeMA ratings were low or (for roflumilast 0.15%) moderate. Downgrades were made for within‐trial bias in all CINeMA judgements, and some were also downgraded for imprecision and heterogeneity. Subgroup and sensitivity analyses, narrative information and analysis of patient‐reported eczema symptoms as a continuous outcome are shown in the full Cochrane review [27] and associated repository https://osf.io/6ujga. In general, these were consistent with the main binary analysis of patient‐reported eczema symptoms.

### 3.2 Clinician‐Reported Eczema Signs

NMA included 32 trials (*n* = 4121) which most commonly reported a ≥75% relative improvement in Eczema Area and Severity Index. Potent TCS (OR 8.15, 95% CI 4.90, 13.57), the TCI tacrolimus 0.1% (OR 8.06, 95% CI 3.30, 19.67) and the JAK inhibitors ruxolitinib 1.5% (OR 7.72, 95% CI 4.92, 12.10) and delgocitinib 0.5% (OR 7.61, 95% CI 3.72, 15.58) were ranked as most effective. Mild TCS (OR 2.22, 95% CI 0.74, 6.64), the PDE‐4 inhibitors roflumilast 0.15% (OR 2.43, 95% CI 0.88, 6.70) and crisaborole 2% (OR 2.98, 95% CI 1.42, 6.26) and the AHR activator tapinarof 1% (OR 2.45, 95% CI 1.00, 6.02) were ranked as least effective (Table 2). Confidence intervals were wide and overlapping for most comparisons, but CINeMA ratings were moderate or high for most licensed interventions. CINeMA downgrades were most commonly made for within‐trial bias, but also imprecision and heterogeneity. Subgroup and sensitivity analyses, narrative information and analysis of clinician‐reported eczema signs as a continuous outcome are shown in the full Cochrane review [27] and associated repository. In general, these were consistent with the main binary analysis of clinician‐reported eczema signs. However, NMA of clinician‐reported eczema signs yielded some counter‐intuitive findings such as increased effectiveness of lower potency TCI and TCS when indirectly compared with higher potency TCI and TCS.

### 3.3 Investigator Global Assessment

NMA included 140 trials (*n* = 23,383) which most commonly reported ‘clear or almost clear’ eczema on a 6‐point Investigator Global Assessment. Potent TCS (OR 5.00, 95% CI 3.80, 6.58), very potent TCS (OR 8.34, 95% CI 4.73, 14.67), the JAK inhibitors ruxolitinib 1.5%, (OR 9.34, 95% CI 4.80, 18.18), delgocitinib 0.5% (OR 10.08, 95% CI 2.65, 38.37) and delgocitinib 0.25% (OR 6.87, 95% CI 1.79, 26.33) and the TCI tacrolimus 0.1% (OR 5.06, 95% CI 3.59, 7.13) were ranked as most effective. Mild TCS (OR 1.38, 95% CI 0.94, 2.02), the PDE‐4 inhibitors roflumilast 0.15% (OR 2.43, 95% CI 0.65, 9.01), crisaborole 2% (OR 2.14, 95% CI 1.22, 3.76), difamilast 0.3% (OR 2.56, 95% CI 1.37, 4.78) and difamilast 1% (OR 3.45, 95% CI 1.97, 6.02) and the TCIs tacrolimus 0.03% (OR 3.53, 95% CI 2.60, 4.80) and pimecrolimus 1% (OR 2.39, 95% CI 1.78, 3.21) were ranked as least effective (Table 3). Confidence intervals were wide and overlapping for most comparisons, and CINeMA ratings were low or moderate for most licensed interventions. CINeMA downgrades were most commonly made for within‐trial bias. Subgroup and sensitivity analyses and narrative information are shown in the full Cochrane review [27] and associated repository. In general, these were consistent with the main IGA analysis. In a sensitivity analysis of low risk of bias data (12 trials, *n* = 1639), potent TCS and the JAK inhibitors delgocitinib 0.5% and delgocitinib 0.25% ranked as most effective, and the TCI pimecrolimus 1%, PDE 4 inhibitors roflumilast 0.15%, difamilast 1% and difamilast 0.3% least effective.

### 3.4 Local Application Site Reactions

NMA included 83 trials (*n* = 18,992) reporting tolerability events, burning, stinging and/or irritation reactions. TCIs tacrolimus 0.1% and 0.03% and pimecrolimus 1% and PDE 4 inhibitor crisaborole 2% were ranked as most likely to cause application site reactions and mild to very potent TCS as least likely (Table 4). Confidence intervals were wide for most comparisons, and CINeMA ratings were low or moderate for most licensed interventions, but high for crisaborole 2%. CINeMA downgrades were most commonly made for within‐trial bias and imprecision. Subgroup and sensitivity analyses and narrative information are shown in the full Cochrane review [27] and associated repository. In general, these were consistent with the main analysis.

### 3.5 Skin Thinning/Atrophy

**FIGURE 4:**
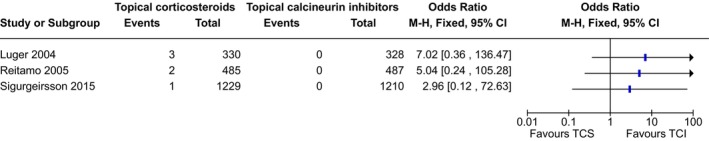
Effect of longer‐term use of topical steroids versus topical calcineurin inhibitors on risk of skin thinning. Summary of trials reporting risk of skin thinning/atrophy with longer term (6–60 months) topical anti‐inflammatory treatments. The trials evaluated potent TCS versus tacrolimus 0.1% over 6 months follow‐up [28], moderate TCS versus pimecrolimus 1% over 1 year follow‐up [29], and mild/moderate TCS versus pimecrolimus 1% over 5 years follow‐up [30]. They were all funded by pimecrolimus or tacrolimus manufacturers and included treatment of both facial and non‐facial areas affected by eczema. Authors did not comment on the location, nature, severity or reversibility of the skin thinning changes identified.

NMA included 25 trials (*n* = 3691, 36 events) reporting skin thinning, atrophy, striae and/or telangiectasia. There was no significant increase in odds of skin thinning/atrophy with mild to very potent TCS or the TCIs tacrolimus 0.1% and pimecrolimus 1% compared with vehicle. CINeMA ratings were low for all comparisons, due to within‐trial bias and imprecision. Subgroup and sensitivity analyses and narrative information are shown in the full Cochrane review [27] and associated repository. In general, these were consistent with the main analysis. Longer‐term data over 6–60 months for this outcome were insufficient for NMA but were reported for TCS versus TCI in three trials (Figure 4), showing an increase in long‐term skin thinning with TCS (6 events in 2044 participants with TCS versus 0 events in 2025 participants with TCI; *p* = 0.031, Fisher's exact test). The three included trials evaluated potent TCS versus tacrolimus 0.1% over 6 months follow‐up [28], moderate TCS versus pimecrolimus 1% over 1‐year follow‐up [29] and mild/moderate TCS versus pimecrolimus 1% over 5 years follow‐up [30]. The three trials were all funded by TCI manufacturers and included treatment of both facial and non‐facial areas affected by eczema. The trial authors did not comment on reversibility of the skin thinning changes identified, nor did they provide details about location and nature of the changes identified.

### 3.6 Other Outcomes

NMA was not possible for health‐related quality of life, long‐term control or longer‐term outcome assessment for any of the above outcomes, due to insufficient data. NMA of pigmentary changes (8 trials of TCS and a PDE‐4 inhibitor, *n* = 1786, 3 events) did not show any significant increase in odds of pigmentation changes compared to vehicle, with low confidence for mild, moderate or potent TCS and moderate confidence for crisaborole 2%. NMA of withdrawal due to short‐term adverse events (11 trials of TCS, TCI, JAK inhibitors and other interventions, *n* = 2404) did not show any significant increase in odds of withdrawal compared to vehicle with any intervention, with low confidence. There was a lack of long‐term safety data.

## 4 Discussion

In this Cochrane systematic review and NMA of topical anti‐inflammatory treatments for eczema, we found potent TCS, JAK inhibitors and the TCI tacrolimus 0.1% were consistently ranked as among the most effective topical anti‐inflammatory treatments for eczema. Our confidence in the findings was low or moderate, largely due to concerns about selective reporting; and we were not able to reliably rank the comparative effectiveness of these three treatments. Safety data were limited, but local application site reactions were most common with tacrolimus 0.1% and the PDE‐4 inhibitor crisaborole 2%. Although skin thinning was not increased with short‐term (<16 weeks) use of any TCS potency, skin thinning was reported as an adverse effect in 0.3% participants treated with TCS for 6 to 60 months.

These findings largely relate to short‐term use of topical anti‐inflammatory treatments for treating non‐severe eczema in adults living in high‐income countries. There is a need for more work to identify longer‐term outcomes, to evaluate effectiveness and safety in young children, in whom eczema is most common, and in low‐middle income countries [1]. For example, recent trials have reported no impact of intermittent mild–moderate TCS on young children's growth over up to 5 years, but significant growth suppression when a proactive potent TCS regimen is used in infants with eczema [31, 32, 33]. We did not formally evaluate cost effectiveness, but it is worthwhile noting that per gram, TCI, JAK inhibitors, PDE‐4 inhibitors and AHR activators typically cost 4–20 times more than TCS.

Our findings are broadly consistent with previous literature, including a recent NMA of topical anti‐inflammatory treatments for eczema [8]. However, there are some differences in emphasis between the two NMA findings, including slightly lower efficacy ranking of JAK inhibitors by Chu et al., and a different focus of adverse event outcomes. Inclusion criteria differed between the two NMAs, and there were some differences in methodology. For example, the review of Chu et al. excluded within‐participant trials, and included complementary therapy, moisturisers, non‐licensed treatment regimens and secondary prevention of eczema flares using regular anti‐inflammatory treatment which were not part of this Cochrane review. This is likely to explain any differences in rankings between the two NMAs, for comparable outcomes. Our finding that TCS rarely cause skin thinning is consistent with the conclusions of a separate Cochrane review evaluating strategies for intermittent short‐term treatment of eczema with TCS [11].

We found significant issues related to risk of bias in this dataset, especially selective reporting. Despite completion and publication of 291 trials with 45,846 participants, there was insufficient low risk of bias information to undertake NMA for key outcomes of interest. Risk of bias is an issue in many other areas of healthcare evidence, and in some specific fields such as infant nutrition, transparent and complete reporting of clinical trial outcomes is almost completely absent from the literature [34, 35]. In contrast to this Cochrane review of eczema treatment trials, a Cochrane review of eczema *prevention* trials using moisturisers, where there is less commercial involvement, found a high level of transparency and complete outcome reporting [36, 37]. In order to effectively build the evidence base to support practice in topical eczema treatment, improved trial registration, transparency and reporting of clinical trials is clearly needed.

Although we identified 291 trials with relevant outcome data, for most networks the majority of trials did not report data that could be included. Individual networks included between 8 and 140 trials each, with other trials either not reporting the relevant outcome or reporting it in such a way that inclusion in NMA was not possible. These additional data are included in the full Cochrane review and associated repository. Incomplete reporting of data was a common feature, with numbers of participants evaluated, means and standard deviations often missing from trial reports. These issues could be effectively resolved by more complete reporting and widespread uptake of core outcome measures in future eczema treatment trials. A further limitation of the dataset analysed is that eczema severity was not reported in a consistent way across studies addressing a range of eczema severities. This means that it was not possible to fully identify and account for potential network intransitivity.

In conclusion, in this NMA of topical anti‐inflammatory treatments for eczema, we found potent TCS, JAK inhibitors and tacrolimus 0.1% were statistically ranked as the most effective short‐term treatments, with varied confidence. Local application site reactions were most common with tacrolimus 0.1% and crisaborole 2%, and skin thinning with TCS treatment was rarely reported. Further work is needed to identify long‐term outcomes associated with these treatments, especially in young children.

## Author Contributions

S.J.L. and R.J.B. co‐ordinated contributions from the co‐authors and wrote the final draft of the review. S.J.L., B.C., C.R., L.S. and R.J.B. screened papers against eligibility criteria. S.J.L., B.C., C.R., R.P., E.A., M.D., M.F. and R.J.B. extracted data for the review. S.J.L., B.C., C.R., R.P., E.A., M.D., M.F. and R.J.B. assessed risk of bias in included trials. E.V.V., B.C. and S.C. entered data into RevMan. E.V.V., S.J.L., B.C., L.S., C.R., B.S., E.A., A.R., D.K.C., M.F., M.S., H.C.W., S.C., A.M.D. and R.J.B. analysed and interpreted data. A.R. was the consumer co‐author and checked the review for readability and clarity, as well as ensuring outcomes were relevant to consumers. E.V.V., B.S., E.A. and S.C. were the statistical and methodological co‐authors. L.S., D.K.C., M.F., M.S., H.C.W., A.M.D. and R.J.B. were the clinical co‐authors.

## Conflicts of Interest

The authors declare the following interests. S.J.L.: NIHR grants, published evidence syntheses and knowledge mobilisation work on the topic and an editorial regarding an included trial. R.P.: Commissioning Editor, The Cochrane Collaboration. E.A.: employed by Cochrane as an Evidence Synthesis Methodology Editor within the Cochrane Methods Support Unit. D.K.C.: author on AAAAI/ACAAI Atopic Dermatitis topical treatments systematic review and guideline. M.F.: payments from Maruho, Otsuka Pharmaceutical, Torii Pharmaceutical; H.C.W.: investigator on an included trial. Suzie Cro: NIHR advanced fellowship. A.M.D.: author of Canadian Dermatology Today; dermatologist at Women's College Hospital; Vice Chair of Scientific and Medical Advisory Committee and research grants from the National Eczema Association. Consultant for Canadian Association for Drugs and Technology in Health; Editor of Cochrane Skin. R.J.B.: fees for editorial work from Wiley and the British Society for Allergy and Clinical Immunology, and for expert witness work in relation to allergy. All other authors declare no conflict of interest.

## Data Availability

The data that support the findings of this trial are available from the corresponding author upon reasonable request. This article is based on a Cochrane Review published in the Cochrane Database of Systematic Reviews [27]. Cochrane Reviews are regularly updated as new evidence emerges and in response to feedback, and the Cochrane Database of Systematic Reviews should be consulted for the most recent version of the review.
